# Effects of Calcium Ion, Calpains, and Calcium Channel Blockers on Retinitis Pigmentosa

**DOI:** 10.1155/2011/292040

**Published:** 2010-12-23

**Authors:** Mitsuru Nakazawa

**Affiliations:** Department of Ophthalmology, Hirosaki University Graduate School of Medicine, 5 Zaifu-cho, Hirosaki 036-8561, Japan

## Abstract

Recent advances in molecular genetic studies have revealed many of the causative genes of retinitis pigmentosa (RP). These achievements have provided clues to the mechanisms of photoreceptor degeneration in RP. Apoptosis is known to be a final common pathway in RP and, therefore, a possible therapeutic target for photoreceptor rescue. However, apoptosis is not a single molecular cascade, but consists of many different reactions such as caspase-dependent and caspase-independent pathways commonly leading to DNA fractionation and cell death. The intracellular concentration of calcium ions is also known to increase in apoptosis. These findings suggest that calpains, one of the calcium-dependent proteinases, play some roles in the process of photoreceptor apoptosis and that calcium channel antagonists may potentially inhibit photoreceptor apoptosis. Herein, the effects of calpains and calcium channel antagonists on photoreceptor degeneration are reviewed.

## 1. Introduction

 Retinitis pigmentosa (RP) represents a group of hereditary retinal degenerations principally characterized by progressive rod-dominant photoreceptor degeneration in the initial stage and eventual cone photoreceptor degeneration in later stages. Patients with RP mainly complain of night blindness and photophobia in the early stage, followed by gradual constriction of the visual field, decreased visual acuity, and color blindness in later stages. The prevalence of RP is roughly 1 in 4,000-5,000 people, and the condition is common in both Asian and Western countries. Significant features of RP include heterogeneity in both clinical and genetic characteristics. For instance, the severity and progression of RP vary from patient to patient even in the same family, despite affected members presumably sharing the same causative gene mutation. Heredities are also heterogeneous, characterized by at least 3 different modes of inheritance, such as autosomal-dominant, autosomal-recessive, and X-linked patterns. Since a mutation in the rhodopsin gene was first identified as causing one type of autosomal-dominant RP [1], at least 48 different causative genes have been identified (RetNet: http://www.sph.uth.tmc.edu/retnet/disease.htm); however, many other putative causative genes and mutations have yet to be identified. Molecular genetic studies have also demonstrated that a primary lesion in RP involves photoreceptor and/or retinal pigment epithelial cells in which many causative genes are specifically expressed under physiological conditions. Photoreceptor or retinal pigment epithelial cells are known to degenerate mostly through apoptosis [2], which is now understood as a final common pathway for RP at the cellular level. As the mechanisms of photoreceptor degeneration have been gradually elucidated, studies on therapeutic approaches have dramatically increased, including pharmacotherapy, cellular transplantation, gene therapy, regenerative therapy, and retinal prosthesis. This paper mainly focuses on studies examining the effects of calcium ions and calpains on photoreceptor apoptosis, as well as pharmacological treatments for RP using calcium channel antagonists.

## 2. Genetic Background of RP

 One of the most important breakthroughs in RP research was the identification of a point mutation (P23H) in the rhodopsin gene as a causative gene mutation for one form of autosomal-dominant RP [1, 3]. Since then, using a candidate gene approach, various mutations in the rhodopsin gene and many other genes have been identified in several RP families. These include mutations in the genes encoding *β*- and *α*-subunits of rod cGMP-phosphodiesterase for autosomal-recessive RP [4, 5] and peripherin/RDS (RDS: retinal degeneration slow) for autosomal-dominant RP [6, 7]. These findings in the early 1990s suggested to many researchers that RP is caused by a single or one allelic pair of mutations in one of the genes specifically or dominantly expressed in photoreceptor cells. The candidate gene approach was also relatively easy to perform once researchers suspected genes already known to be retina specific as possible candidates for RP. Many other genes and mutations in these genes were then found to cause RP (Table 1). However, the candidate gene approach is limited in that screening can only be performed for known genes and involvement could not be ascertained for previously unknown genes that might be expressed not only in the retina, but also in other organs or tissues in a ubiquitous fashion. For these reasons, genetic linkage and/or association analyses have been performed in combination with a candidate gene approach to identify many other previously unpredictable genes as causative genes for RP. This group includes *PRPF31* [8], *PRPF3* [9], *PRPF8* [10], *IMPDH1* [11], *Mertk *[12, 13], and *CA4* [14] which are expressed in other tissues besides retina (Table 1). These findings indicate that photoreceptors and retinal pigment epithelium are much more active in protein synthesis than any other tissues and show high levels of gene expression and protein metabolism. In addition, molecular genetic studies have disclosed that RP is genetically more heterogeneous than it used to be considered and that the genetic heterogeneity may be one explanation for the clinical heterogeneity.

## 3. Photoreceptor Apoptosis as a Common Mechanism in RP

 Despite the clinical and genetic heterogeneity, RP demonstrates common features derived from rod-predominant degeneration. This essential phenomenon allowed researchers to suspect some common mechanisms leading to photoreceptor cell death once the patient carries a single or one allelic pair of many causative gene mutations. Apoptosis is a genetically programmed mechanism that leads cells to death, and RP has been known to be initiated by photoreceptor apoptosis as a final common pathway at the cellular level, irrespective of gene mutations. For instance, apoptosis was detected in retinal degeneration 1 (rd1), rds, and rhodopsin mutant mice [2]. To date, many pathways have been found for apoptosis itself, involving caspases, cathepsins, calpains, apoptosis-inducing factor (AIF), Fas, and more. Once abnormal and/or insufficient structural or metabolic stresses induced by a certain gene mutation exceed predetermined thresholds that a cell can tolerate, mechanisms of apoptosis are initiated that lead to nuclear DNA fragmentation and subsequent cell death. Many experimental studies have supported that caspase-dependent or -independent apoptotic pathways are activated during experimental retinal degeneration models [15, 16]. Apoptosis can thus be considered as a therapeutic target as it plays many roles in retinitis pigmentosa [17, 18].

 Calpains [EC 3.4.22.17], a group of calcium-dependent cysteine proteases, play some important roles in caspase-independent photoreceptor apoptotic pathways with light-induced retinal damage [19] and in rd1 mice [20, 21] and Royal College of Surgeons (RCS) rats [22] as models of retinal degeneration. Calpains are also involved in calcium-induced cell death in a murine photoreceptor-derived cell line [23, 24]. There is little doubt that intracellular concentrations of calcium ion were elevated in apoptosis [25–28]. As calcium influx is actually elevated in degenerating rd1 rod photoreceptors [20, 29], calpains are suspected to play important roles in photoreceptor apoptosis in RP. In addition, calpain inhibitors and calcium channel blockers appear to offer reasonable candidates at least in part as pharmacotherapeutic agents for RP. Transient inhibitory effects of calpain inhibitors on photoreceptor apoptosis in RCS rats have recently been described by Mizukoshi et al. [22].

## 4. Effects of Calcium Ion on Photoreceptor Apoptosis

**Figure 1:**
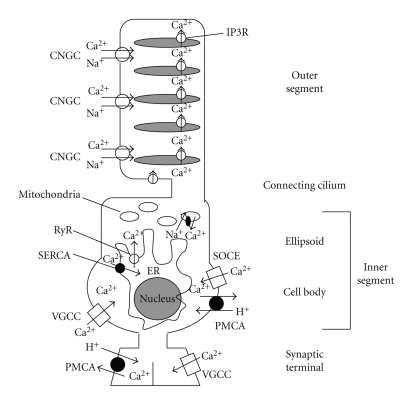
Schematic view of calcium channels and transporters in the rod-photoreceptor. Abbreviations: CNGC, cyclic-nucleotide-gated cation channel; VGCC: voltage-gated calcium channel; PMCA: plasma membrane calcium ATPase; SOCE: store-operated calcium entry; ER: endoplasmic reticulum; SERCA: sarcoplasmic-endoplasmic reticulum Ca^2+^-ATPase; IP3R: inositol 1, 4, 5-triphoaphate receptor; RyR: ryanodine receptor. Ca^2+^  stock and release from the mitochondria are mediated by Ca^2+^  uniporter channels and Na^+^/Ca^2+^  transporters.

 As mentioned above, intracellular concentrations of calcium ion are increased in apoptosis [20, 25–29]. Intracellular calcium ions are provided through several types of calcium channels and transporters located on cell membranes, endoplasmic reticulum, and mitochondria. Cyclic-nucleotide-gated cation channels (CNGCs) are located in the outer segment and closed by depletion of cGMP as a result of the phototransduction reaction triggering hyperpolarization. L-type voltage-gated calcium channels (VGCCs) are located in the cell body and synaptic terminal and are closed by hyperpolarization of the cell membrane induced by phototransduction. Steele Jr. et al. [30] suggested that the average concentration of calcium in the terminal ranges from *∼*350 nM in hyperpolarized light-adapted cells to more than 39 *μ*M in cells depolarized to dark potentials in salamander rods and cones. In addition to CNGC and VGCC, intracellular concentrations of calcium ions are regulated by many other factors, such as plasma membrane calcium ATPase, store-operated calcium entry, calcium stores in the endoplasmic reticulum, and mitochondria (Figure 1). Under pathological conditions, like those in rd1 mice, intracellular calcium levels significantly increase in rods, even before the detection of apoptotic cells [29]. The marked elevation of intracellular concentrations of calcium ions activates downstream reactions, including hydrolytic enzymes like calpains, and eventually leads to cell death [25]. Excessive calcium influx is initiated in the cytosol and subsequently in mitochondria in rd1 mouse [29], suggesting that increased calcium ions may affect many biochemical cascades and reactions not only in the cytosol but also in the mitochondria [31, 32]. As mentioned above, increased intracellular calcium concentrations activate calpains, leading to the activation of both caspase-dependent and -independent apoptotic pathways. First, as a caspase-dependent pathway, calpains activate caspase 12, which sequentially activates caspases 9, 3, 4, and 7 and finally apoptosis is upregulated. Second, cytosolic calpains further activate cathepsins and mitochondrial calpains activate AIF, which subsequently translocates from mitochondria to the nucleus [22]. This reaction has been speculated to represent one of the caspase-independent pathways of apoptosis [33, 34].

## 5. Ca^2+^ Channel Antagonists for Photoreceptor Apoptosis in Animal Experiments

 Since Frasson et al. [35] first reported the effects of D-cis-diltiazem, a benzothiazepin calcium channel antagonist which blocks both CNGC and VGCC, on photoreceptor protection in rd1 mice, several investigators have reported positive and negative effects of calcium channel blockers on animal models of RP [19–21, 36–43]. Since rd1 is caused by a mutation in the gene encoding the *β*-subunit of rod cGMP-phosphodiesterase, one of the key enzymes in the phototransduction pathway, CNGCs located in the outer segment cannot be closed despite light stimulation in the rod photoreceptor cells. Inhibition of light-induced hyperpolarization, caused by a mutation in the rod cGMP-phosphodiesterase gene, also does not close VGCC. These phenomena increase calcium influx in both outer and inner segments in rd1 mice. The intracellular concentration of calcium ions is subsequently elevated, leading to photoreceptor apoptosis [35], possibly by upregulation of calpains and other proteins [28]. Sanges et al. [20] demonstrated that systemic administration of D-cis-diltiazem reduced intracellular concentrations of calcium, downregulating calpains and photoreceptor apoptosis in rd1 mice. Direct inhibitory effects of D-cis-diltiazem on CNGC [44] or L-type VGCC [39] have been reported, and D-cis-diltiazem effectively blocks photoreceptor light damage in mouse models by inhibiting photoreceptor apoptosis [19]. In contrast, L-cis isomer inhibits L-type VGCC similarly to D-cis isomer [45]. The difference in action between D-cis- and L-cis-diltiazems on photoreceptor neuroprotection [35] suggests that CNGC might also be important for photoreceptor neuroprotection [44]. Read et al. [46] also reported that the *β*-subunit of VGCC knock-out rd1 mice showed retardation of photoreceptor degeneration, suggesting that blockage of calcium influx may partially contribute to photoreceptor rescue in these animal models although it did not prevent photoreceptor degeneration. Despite these studies, however, Pawlyk et al. [36] and Takano et al. [41] found no rescue effects of D-cis-diltiazem on retinal degeneration in rd1 mice, and Bush et al. [42] also reported that D-cis-diltiazem was ineffective for photoreceptor rescue in rhodopsin P23H transgenic rats. 

 While the effects of diltiazem on animal models of retinal degeneration remain controversial, another type of calcium channel blocker, nilvadipine, a member of the dihydropyridine derivatives, is another candidate therapeutic agent for RP. Nilvadipine has low-voltage-activated calcium blocking actions in addition to L-type high-voltage calcium blocking actions. The hydrophobic nature induced by the chemical structure of nilvadipine allows high permeability to the central nervous system, including the retina [47]. Systemic administration of nilvadipine has been shown to be effective for protecting photoreceptors in RCS rats [37, 40], rd1 mice [41], and heterozygous rd2 (rds) mice [43]. In addition to direct effects of calcium channel blockers on intracellular concentrations of calcium ion in photoreceptor cells, other indirect effects are expected such as increased expression of fibroblast growth factor (FGF) 2 [40, 41] and ciliary neurotrophic factor (CNTF) [43] in the retina, and increased choroidal blood flow [48]. Since FGF2 and CNTF are known to exert photoreceptor-protective effects [49–56], upregulating such intrinsic neurotrophic factors by nilvadipine may demonstrate beneficial effects against RP. CNTF has also been applied as a clinical trial for RP [57]. In addition, oxidative stress may be involved in photoreceptor death in RP [58–63], and nilvadipine has the highest antioxidant potency among calcium channel blockers [64]. The direct effects of calcium channel blockers on photoreceptor calpains have not yet been studied. Studies involving calcium channel antagonists are listed in Table 2. As the effects of calcium channel blockers on photoreceptor rescue remain controversial, further biochemical studies are required in order to facilitate our understanding of the mechanisms of photoreceptor degeneration induced by various types of gene mutations, the effects of intracellular calcium ions on downstream reactions, and the effects of calcium channel blockers on both concentrations of calcium ions and downstream reactions in various types of heterogeneous conditions of RP. Although human RP is caused by various kinds of heterogeneous causative gene mutations, our understanding regarding photoreceptor degeneration in RP is still limited to relatively small numbers of experimental models of RP.

## 6. Human Trials

 Although human RP is genetically heterogeneous, possible rescue effects of calcium channel blockers on photoreceptor degeneration in certain animal models of RP, such as rd1 and rds mice and RCS rats, have encouraged researchers to expect therapeutic effects of calcium channel blockers for RP. Pasantes-Morales et al. [65] reported that a combination of D-cis-diltiazem, taurin, and vitamin E has beneficial effects on the visual field progression, although the study did not clarify whether diltiazem alone demonstrated beneficial effects. Ohguro [66] reported the photoreceptor rescue effects of nilvadipine in a small patient group. We expanded his nilvadipine study for RP patients to confirm the results. Although both treated and control groups are still small, our results have shown significant retardation of the mean deviation (MD) slope as calculated by the central visual field (Humphry Visual Field Analyzer, 10-2 Program) after a mean of 48 months of observation [67]. As these pilot studies are small-sized and cannot completely exclude possible biases, a large-scale, randomized, multicenter human trial of calcium channel blockers is required in order to evaluate their efficacy as therapeutic agents for RP.

## 7. Future Insights

 As pharmacotherapeutic agents for RP, vitamin A [68, 69] and lutein [70] are reportedly effective in slowing RP, and carbonic anhydrase inhibitors appear effective for reducing chronic cystoid macular edema [71, 72], although the basic molecular mechanisms underlying these actions remain unclear. Effects of calcium channel blockers have been speculated based on the molecular mechanisms in RP identified in recent molecular genetic [4] and animal studies [20, 35] of RP and also research on neuroprotection for glaucoma [73]. In addition to previous pilot studies, large-scale human trials to examine the effects of calcium channel blockers in the progression of RP are needed to obtain solid evidence-based results. Since calcium channel blockers may not effectively block enough calcium influx to rescue degenerating photoreceptors depending on the kinds of gene mutations, downstream reactions like calpains should be considered when planning therapy. Effects of calpain inhibitors on human RP patients should also be examined in the future. As other modern technologies have advanced, new therapeutic modalities including gene therapy, retinal prostheses, and regenerative medicine have become increasingly developed, and some applications of these technologies are now commercially available. Of note is the fact that pharmacotherapeutic agents aimed at photoreceptor rescue can be used in combination with gene therapy and regenerative medicine.

## Figures and Tables

**Table 1:** List of causative genes of RP: retina specific and nonspecific.

Category	ADRP (20)	ARRP (25)	XLRP (2)
Retina specific	CRX	ABCA4	RP2
	FSCN2	CERKL	
	GUCA1B	CNGA1	
	NRL	CNGB1	
	NR2E3	CRB1	
	PRPH2	EYES	
	RDH12	IDH3B	
	RHO	LRAT	
	ROM1	NR2E3	
	RP1	NRL	
	RP9	PDE6A	
	SEMA4A	PDE6B	
		PRCD	
		PROM1	
		RBP3	
		RGR	
		RHO	
		RLBP1	
		RP1	
		RPE65	
		SAG	
		SPATA7	
		TUP1	
		USH2	

Retina nonspecific	CA4	MERTK	RPGR
	IMPDH1		
	KHLH7		
	PRPF3		
	PRPF8		
	PRPF31		
	SNRNP200		
	TOPORS		

Abbreviations are listed in Ret:Net: http://www.sph.uth.tmc.edu/retnet/ disease.htm.

**Table 2:** Photoreceptor rescue by calcium channel antagonists.

	Authors	Year	Ref.
Supportive			
Diltiazem on rd1 mouse	Frasson et al.	1999	[35]
Diltiazem on rd1 mouse	Sanges et al.	2006	[20]
Diltiazem on light damage	Vallaza-Deschamps et al.	2005	[19]
Calcium channel knockout on rd1	Read et al.	2002	[46]
Nilvadipine on rd1 mouse	Takano et al.	2004	[41]
Nilvadipine on RCS rat	Yamazaki et al.	2002	[37]
Nilvadipine on RCS rat	Sato et al.	2003	[40]
Nilvadipine on rd2 (rds) mouse	Takeuchi et al.	2008	[43]
Negative			
Diltiazem on rd1 mouse	Pawlyk et al.	2002	[36]
Diltiazem on rd1 mouse	Takano et al.	2004	[41]
Diltiazem on pde *β* knock-out dog	Pearce-Kelling et al.	2001	[21]
Diltiazem on rhodopsin P23H rat	Bush et al.	2000	[42]
